# Preclinical Evaluation of the Copper-64 Labeled GRPR-Antagonist RM26 in Comparison with the Cobalt-55 Labeled Counterpart for PET-Imaging of Prostate Cancer

**DOI:** 10.3390/molecules25245993

**Published:** 2020-12-18

**Authors:** Christina Baun, Bogdan Mitran, Sara S. Rinne, Johan H. Dam, Birgitte B. Olsen, Vladimir Tolmachev, Anna Orlova, Helge Thisgaard

**Affiliations:** 1 PET & Cyclotron Centre, Department of Nuclear Medicine, Odense University Hospital, 5000 Odense, Denmark; Johan.Dam@rsyd.dk (J.H.D.); birgitte.brinkmann.olsen@rsyd.dk (B.B.O.); Helge.Thisgaard@rsyd.dk (H.T.); 2 Department of Clinical Research, University of Southern Denmark, 5000 Odense, Denmark; 3 Department of Medicinal Chemistry, Faculty of Pharmacy, Uppsala University, 75183 Uppsala, Sweden; bogdan.mitran@ki.se (B.M.); sara.rinne@ilk.uu.se (S.S.R.); anna.orlova@ilk.uu.se (A.O.); 4 Department of Immunology, Genetic and Pathology, Uppsala University, 75185 Uppsala, Sweden; vladimir.tolmachev@igp.uu.se; 5 Research Centrum for Oncotheranostics, Research School of Chemistry and Applied Biomedical Sciences, Tomsk Polytechnic University, 634050 Tomsk, Russia; 6 Science for Life Laboratory, Department of Medicinal Chemistry, Uppsala University, 75237 Uppsala, Sweden

**Keywords:** gastrin-releasing peptide receptor, RM26, NOTA, NODAGA, bombesin antagonist, Co-55, Cu-64, PET-imaging, prostate cancer

## Abstract

Gastrin-releasing peptide receptor (GRPR) is overexpressed in the majority of prostate cancers. This study aimed to investigate the potential of ^64^Cu (radionuclide for late time-point PET-imaging) for imaging of GRPR expression using NOTA-PEG_2_-RM26 and NODAGA-PEG_2_-RM26. Methods: NOTA/NODAGA-PEG_2_-RM26 were labeled with ^64^Cu and evaluated in GRPR-expressing PC-3 cells. Biodistribution of [^64^Cu]Cu-NOTA/NODAGA-PEG_2_-RM26 was studied in PC-3 xenografted mice and compared to the biodistribution of [^57^Co]Co-NOTA/NODAGA-PEG_2_-RM26 at 3 and 24 h p.i. Preclinical PET/CT imaging was performed in tumor-bearing mice. NOTA/NODAGA-PEG_2_-RM26 were stably labeled with ^64^Cu with quantitative yields. In vitro, binding of [^64^Cu]Cu-NOTA/NODAGA-PEG_2_-RM26 was rapid and GRPR-specific with slow internalization. In vivo, [^64^Cu]Cu-NOTA/NODAGA-PEG_2_-RM26 bound specifically to GRPR-expressing tumors with fast clearance from blood and normal organs and displayed generally comparable biodistribution profiles to [^57^Co]Co-NOTA/NODAGA-PEG_2_-RM26; tumor uptake exceeded normal tissue uptake 3 h p.i.. Tumor-to-organ ratios did not increase significantly with time. [^64^Cu]Cu-NOTA-PEG_2_-RM26 had a significantly higher liver and pancreas uptake compared to other agents. ^57^Co-labeled radioconjugates showed overall higher tumor-to-non-tumor ratios, compared to the ^64^Cu-labeled counterparts. [^64^Cu]Cu-NOTA/NODAGA-PEG_2_-RM26 was able to visualize GRPR-expression in a murine PC model using PET. However, [^55/57^Co]Co-NOTA/NODAGA-PEG_2_-RM26 provided better in vivo stability and overall higher tumor-to-non-tumor ratios compared with the ^64^Cu-labeled conjugates.

## 1. Introduction

Prostate cancer (PC) is the most frequently diagnosed cancer among men in developed countries and was estimated to be associated with 359,000 deaths worldwide in 2018 [1,2]. Current treatment includes radical prostatectomy and anti-androgen treatment strategies, depending on the primary diagnosis and the staging [3,4]. Initial diagnoses rely on PSA-screening and biopsies, and TNM staging is evaluated by contrast-enhanced CT or MRI, although both modalities have shown a low sensitivity for detection of lymph node metastases [5]. It can be difficult to detect small metastases especially in the pelvis; due to their size they can be overlooked by conventional imaging [5]. Early and accurate staging is important for optimal treatment strategies in the effort to prolong patient survival and to improve the quality of life [3,4]. Positron emission tomography (PET) using different radiopharmaceuticals (including small molecules [^18^F]F-FDG, [^11^C]C-/[^18^F]F-choline, [^11^C]C-acetate, [^18^F]F-FACBC, and anti-PSMA monoclonal antibodies and small molecule ligands) for imaging of PC in combination with anatomical modalities has been found useful in the detection of biochemical relapse, recurrence, and extent of PC lesions [6,7,8]. The PSMA-targeting small molecule ligands were the best among the above-mentioned probes. However, due to heterogeneous PSMA expression in PC the imaging sensitivity is still suboptimal [7]. This emphasizes the need for new diagnostic and therapeutic approaches for PC.

Molecular imaging of gastrin-releasing peptide receptor (GRPR) expression in PC provides an additional opportunity for accurate detection of lesions [9,10]. Several studies have documented the overexpression of GRPR in PC in both primary tumors and metastases at an early stage [11,12,13,14]. Radiolabeling of peptides such as bombesin receptor antagonists for tumor targeting can provide an advantage in receptor-targeting imaging and therapy due to their high affinity for the targeted receptor and the fast clearance from non-targeted tissues [15,16]. Numerous GRPR agonists, and antagonists labeled with radioisotopes have been evaluated for imaging using PET and SPECT and therapy of PC [9,17,18,19]. GRPR antagonists have shown an advantage compared to agonists due to better tumor activity uptake and retention and fast clearance of the tracer from non-targeted tissue, along with a minimum of side effects [19,20,21,22].

We previously evaluated the GRPR antagonist RM26 (D-Phe-Gln-Trp-Ala-Val-Gly-His-Sta-Leu-NH2) conjugated to a variety of chelators and linkers and the derivatives were labeled with a series of radionuclides, e.g., ^111^In for SPECT imaging and ^68^Ga and ^55^Co for PET imaging [23,24]. We demonstrated among other things that, despite high and specific tumor uptake, different macrocyclic chelators’ charges and structures have a strong influence on the binding affinity of the GRPR-targeting peptide and activity retention time, both in tumors and normal tissues [25,26,27,28]. In the effort to optimize tumor-targeting, several groups investigated the labeling of a GRPR antagonist with long-lived radionuclides (reviewed in [10,29]). Next day PET imaging might improve the tumor-to-background ratio and thus, imaging contrast. This may enable the detection of low abdominal lymph node involvement, which requires the highest possible sensitivity and is the ultimate goal in PC imaging [15,16].

A positron-emitting radiometal with a half-life of 10 to 20 h would be optimal for next day imaging. Suitable PET radionuclides for such purposes include ^64^Cu (*T*_1/2_ = 12.7 h), ^86^Y (*T*_1/2_ = 14.7 h), and ^55^Co (*T*_1/2_ = 17.5 h). ^64^Cu has recently gained appreciable attention despite its low positron abundance (17.4%) and has even been suggested as a theranostic isotope with PET-imaging and radiotherapy capabilities [30,31]. The coordination chemistry of copper allows for chelation at room temperature with a wide variety of chelator systems [31]. The NOTA chelator has shown to be superior compared to many other chelators for the labeling of imaging probes with copper [32,33]. Similar to ^55^Co, no-carrier-added ^64^Cu can be produced on a low-energy biomedical cyclotron by the nuclear reaction ^64^Ni(p,n)^64^Cu which allows for local production at hospitals [30]. ATSM labeled with ^64^Cu for visualization of tumor hypoxia and [^64^Cu]Cu-DOTATATE for imaging of neuroendocrine tumors are already in clinical use [34,35]. Further development of radiocopper-labeled ligands for imaging and therapy is of high clinical interest [36].

**Figure 1:**
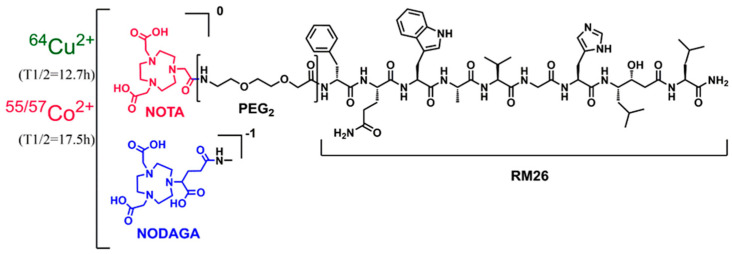
Schematic overview of the structure of [^64^Cu]Cu- and [^55^Co]Co-labeled X-RM26 (X = NOTA-PEG_2_, NODAGA-PEG_2_).

We recently investigated the GRPR-targeting ligands NOTA-, NODAGA-, DOTA-, and DOTAGA-PEG_2_-RM26 labeled with the PET radionuclide ^55^Co, which has shown promising imaging characteristics for next day imaging [24]. In our effort to find the optimal bombesin antagonist for clinical translation, this study aimed to investigate the potential of ^64^Cu for PET-imaging of GRPR expression using the GRPR-targeting ligands NOTA-PEG_2_-RM26 and NODAGA-PEG_2_-RM26. Furthermore, we compared them head-to-head with their earlier reported ^55/57^Co-labeled counterparts (Figure 1) in a preclinical PC-model.

## 2. Results

### 2.1. Labeling, Stability, and In Vitro Characterization of [^64^Cu]Cu-NOTA/NODAGA-PEG_2_-RM26

NOTA-PEG_2_-RM26 and NODAGA-PEG_2_-RM26 were successfully labeled with ^64^Cu with yields and purities exceeding 98% for a molar activity of 4 MBq/nmol and 96% for 40 MBq/nmol. The compounds were stable in serum and excess of ethylenediaminetetraacetic acid (EDTA) with minimal release of free radiocopper (Table 1).

**Figure 2:**
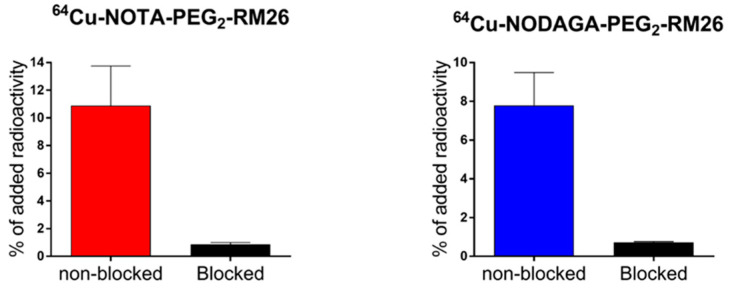
Binding specificity of [^64^Cu]Cu-X-RM26 (X = NOTA-PEG_2_, NODAGA-PEG_2_) to gastrin-releasing peptide receptor (GRPR)-expressing PC-3 cells. Blocked dishes were pre-saturated with 300-fold excess non-labeled conjugates. Cell-associated activity was calculated as a percentage of total added activity. Data are presented as average ± standard deviation.

The in vitro binding specificity assay demonstrated specific binding of [^64^Cu]Cu-X-RM26 (X = NOTA-PEG_2_, NODAGA-PEG_2_) to GRPR-expressing PC-3 cells (Figure 2). The pre-saturation of receptors by addition of a large molar excess of non-labeled peptide caused a significant reduction of [^64^Cu]Cu-X-RM26 uptake.

**Figure 3:**
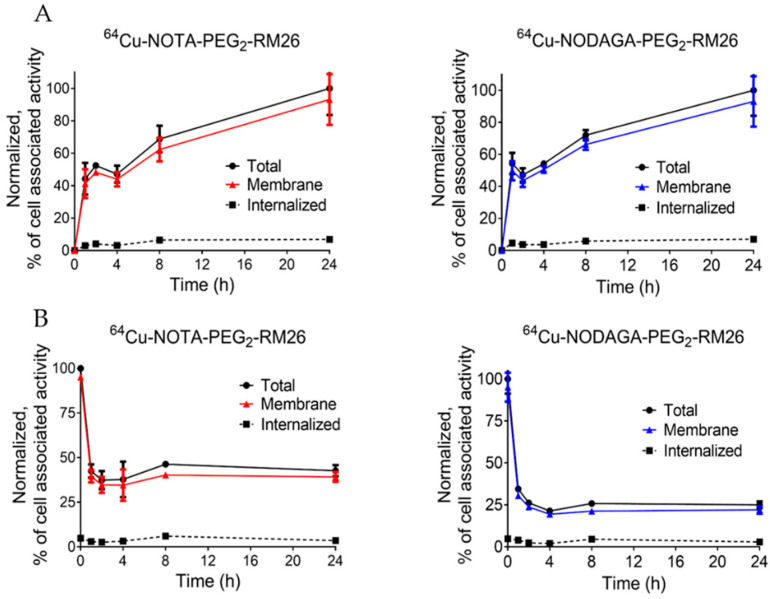
Binding and cellular processing of [^64^Cu]Cu-X-RM26 (X = NOTA-PEG_2_, NODAGA-PEG_2_) by GRPR-expressing PC-3 cells during (**A**) continuous incubation with 1 nM of [^64^Cu]Cu-X-RM26 and (**B**) after interrupted incubation with 1 nM of [^64^Cu]Cu-X-RM26. Cell-bound activity is normalized to the maximum uptake. Data are presented as mean value ± standard deviation. Error bars that are smaller than symbols may not be visible.

Results concerning cellular processing and internalization of [^64^Cu]Cu-X-RM26 are presented in Figure 3A. Cellular processing was similar for both conjugates. Cell-associated activity increased continuously over time, while the internalized fraction was very low at all time points, below 7% of total cell-associated activity. The cellular retention of [^64^Cu]Cu-X-RM26 (Figure 3B) revealed a rapid initial dissociation phase (up to 1 h) followed by a plateau. [^64^Cu]Cu-NOTA-PEG_2_-RM26 had significantly higher retention in cells compared to [^64^Cu]Cu-NODAGA-PEG_2_-RM26 (43 ± 3% of cell-associated activity for NOTA-PEG_2_-RM26 vs 25 ± 2% for NODAGA-PEG_2_-RM26 after 24 h incubation).

**Figure 4:**
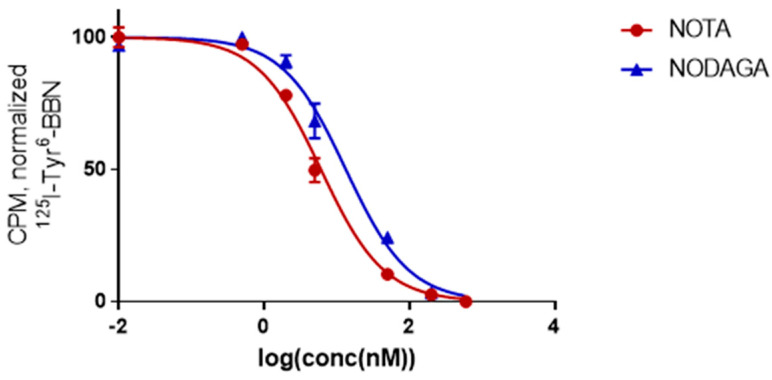
Inhibition of ^125^I-Tyr^4^-BBN binding to PC-3 cells with ^nat^Cu-X-RM26 [X = NOTA-PEG_2_ (●) and NODAGA-PEG_2_ (▲)]. Data are presented as mean value ± standard deviation.

The IC_50_ values were in the low nanomolar range for both conjugates although pronounced chelator-dependent differences could be observed (Figure 4). The IC_50_ for ^nat^Cu-NODAGA-PEG_2_-RM26 showed a two-fold worse binding affinity (12.0 ± 1.0 nM) compared to ^nat^Cu-NOTA-PEG_2_-RM26 (6.1 ± 0.8 nM).

### 2.2. In Vivo Characterization of [^64^Cu]Cu-NOTA/NODAGA-PEG_2_-RM26 and Comparison with ^55/57^Co-Labeled Counterparts

Biodistribution of [^64^Cu]Cu-X-RM26 (X = NOTA-PEG_2_, NODAGA-PEG_2_) was evaluated in mice bearing PC-3 xenografts at 3 and 24 h p.i. For comparison, mice were co-injected with the ^57^Co-labeled counterparts. ^57^Co was used as a convenient surrogate isotope for ^55^Co in the biodistribution part, due to their chemical identity. It was previously demonstrated that ^57^Co could be used for preclinical evaluation of radioagents that are designed to be used with ^55^Co for PET [28]. [^55^Co]Co-X-RM26 has previously shown a remarkable potential for PET imaging of GRPR expression [28].

[^64^Cu]Cu- and [^57^Co]Co-labeled X-RM26 displayed comparable biodistribution profiles with a fast clearance from blood and normal organs, including excretory organs (Table 2). Tumor uptake at 3 h p.i. exceeded the uptake in normal organs for all conjugates. No significant difference was observed in tumor uptake between either the ^64^Cu- and ^57^Co-labeled conjugates or between the different chelators at both time points. Tracer uptake was also observed in GRPR expressing organs, excretory organs, and the gastrointestinal (GI) tract (Table 2).

Several notable differences could be observed between ^64^Cu-labeled and ^57^Co-labeled conjugates. The clearance of activity from blood was significantly slower for [^64^Cu]Cu-NOTA-PEG_2_-RM26 in comparison to both [^64^Cu]Cu-NODAGA-PEG_2_-RM26 and [^57^Co]Co-X-RM26 at both time-points (all *p*-values < 0.0001). The uptake in liver tissue was also significantly higher for ^64^Cu-labeled conjugates at both time-points in comparison to the ^57^Co-labeled counterparts (ten-fold for [^64^Cu]Cu-NOTA-PEG_2_-RM26 and four-fold for [^64^Cu]Cu-NODAGA-PEG_2_-RM26, at 3 h p.i.) (Table 2).

At 3 h p.i. [^64^Cu]Cu-NODAGA-PEG_2_-RM26 showed significantly lower uptake in the GRPR-expressing pancreas, stomach, and small intestines, compared with [^64^Cu]Cu-NOTA-PEG_2_-RM26. For the GI tract, both the ^64^Cu- and ^57^Co-labeled NOTA conjugates showed lower uptake compared with their NODAGA labeled counterparts 3 h p.i. where [^57^Co]Co-NOTA-PEG_2_-RM26 had the lowest activity uptake in comparison with [^64^Cu]Cu-X-RM26. At 24 h p.i. activity uptake in GRPR-expressing tissue (e.g pancreas, stomach, small intestine, and GI tract) decreased and was lower for ^57^Co-labeled conjugates than for ^64^Cu-labeled ones. [^64^Cu]Cu-NOTA-PEG_2_-RM26 showed significantly higher uptake in pancreas at 3 and 24 h p.i. both in comparison with [^64^Cu]Cu-NODAGA-PEG_2_-RM26, and [^57^Co]Co-NOTA-PEG_2_-RM26. At 24 h p.i., [^64^Cu]Cu-NOTA-PEG_2_-RM26 still had the highest uptake among all four conjugates, but not to a significant level. For kidneys at 3 h p.i., [^64^Cu]Cu-NOTA-PEG_2_-RM26 showed significantly lower uptake compared with [^64^Cu]Cu-NODAGA-PEG_2_-RM26 (*p* < 0.0001). The same was observed for the ^57^Co-labeled conjugates, where NOTA-PEG_2_-RM26 also showed significantly lower uptake (*p* = 0.01). At 24 h p.i. the kidney uptake was evened out and no difference was observed between the four different conjugates. For muscle and bone, no significant difference was observed between the four conjugates at both time-points (see Table 2 and Figure S1 in Supplementary Materials).

**Figure 5:**
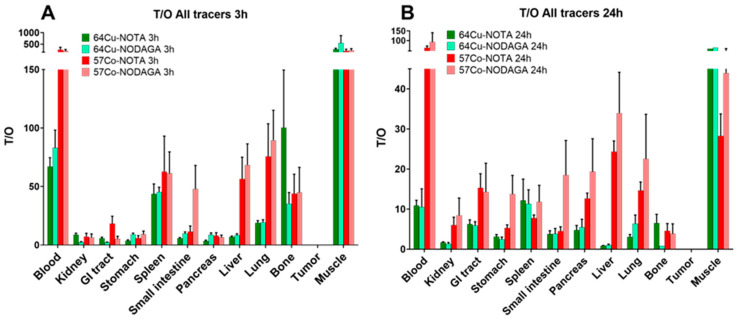
Tumor-to-organ ratios at (**A**) 3 h and (**B**) 24 h p.i. of [^64^Cu]Cu-X-RM26 (X = NOTA-PEG_2_, NODAGA-PEG_2_) in PC-3 xenografted BALB/C nu/nu mice.

The overall higher activity uptake in normal organs resulted in lower tumor-to-blood, tumor-to-liver, and tumor-to-lung ratios for [^64^Cu]Cu-X-RM26 compared with the ^57^Co-labeled counterparts at both time-points (Figure 5 and Table S1). Tumor-to-blood ratios for [^64^Cu]Cu-X-RM26 were three-fold lower compared with [^57^Co]Co-X-RM26 at 3 h p.i., but without reaching statistical significance. At 24 h p.i., the tumor-to-organ ratios were generally higher for [^57^Co]Co-X-RM26 than for [^64^Cu]Cu-X-RM26, except for tumor-to-muscle ratios which were non-significantly higher for [^64^Cu]Cu-X-RM26 compared with [^57^Co]Co-X-RM26 at 3 h (Figure 5A) and 24 h (Figure 5B).

### 2.3. Imaging

**Figure 6:**
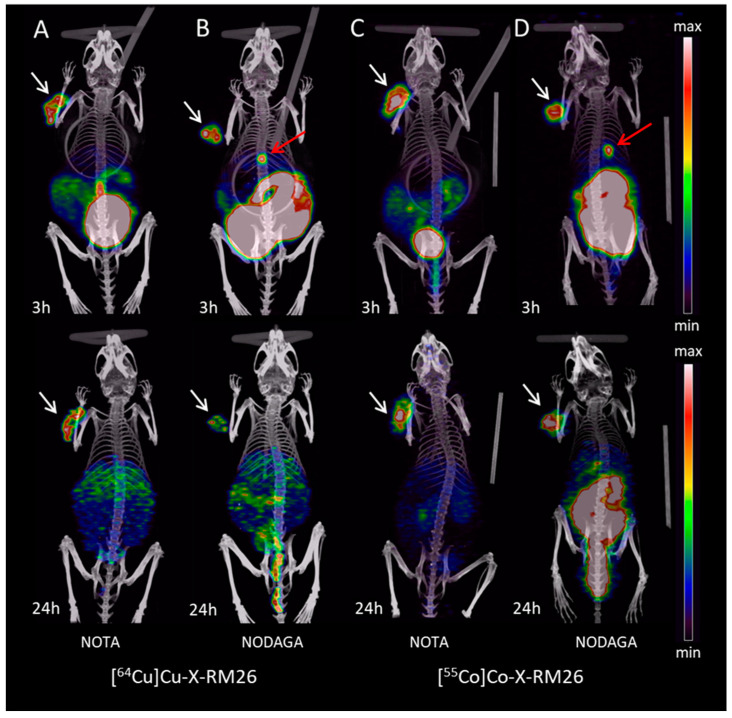
Coronal maximum intensity projection (MIP) preclinical positron emission tomography (PET)/CT images showing tracer distribution in PC-3 xenografted NOD-SCID mice. Uptake and distribution of [^64^Cu]Cu-X-RM26 are shown in images (**A**) and (**B**) and for [^55^Co]Co-X-RM26 in images (**C**) and (**D**), (X = NOTA-PEG_2_ or NODAGA-PEG_2_). The mice were scanned at 3 h (top row) and 24 h (bottom row) p.i. The tumor is shown by the white arrow. The gall bladder was visual in both the 3 h NODAGA scans and is shown by the red arrow. The intensity of PET is displayed from zero to maximum of the tumor uptake.

PET/CT scans of PC-3 tumor-bearing mice injected with [^64^Cu]Cu-X-RM26 or [^55^Co]Co-X-RM26 (X = NOTA-PEG_2_, NODAGA-PEG_2_) are shown as coronal maximum intensity projection (MIP) images in Figure 6. The GRPR expression was successfully visualized in the scans obtained at 3 and 24 h p.i. and confirmed the respective findings from the biodistribution. A chelator-dependent difference was seen for both the ^55^Co- and ^64^Cu-labeled conjugates at 3 h p.i., where NODAGA containing conjugates showed increased uptake in the GI tract in comparison with the NOTA-labeled counterparts. Furthermore, NODAGA conjugates showed clear visualization of the gall bladder at 3 h p.i., presumably due to hepatobiliary excretion of the conjugates. At the later time-point of 24 h p.i., the clearance of activity from normal organs (except for [^55^Co]Co-NODAGA-PEG_2_-RM26) resulted in high imaging contrast for both [^64^Cu]Cu-X-RM26 and [^55^Co]Co-NOTA-PEG_2_-RM26.

## 3. Discussion

Development of radiopharmaceuticals targeting GRPR in PC has gained increasing interest in the effort to improve the diagnostic accuracy by detection of metastases and to develop new treatment strategies [37,38,39]. Patients suffering from PC represent a very large group and even though the tumor is slowly growing in most cases, early and accurate diagnosis is essential for prognosis and the overall survival [3,4]. Radiolabeling of various GRPR antagonists has been investigated with several radionuclides and different chelators to optimize the pharmacokinetics for molecular imaging and targeted radionuclide therapy in PC [40]. The use of long-lived PET-isotopes has shown promising results due to the possibility of late time-point imaging with improved tumor-to-background ratios and thus, image contrast [28].

It is well known, that the charge and geometry of the radiometal-chelator complex can have a major influence on the pharmacokinetic properties of labeled peptides [41,42,43]. Especially for ^64^Cu-labeled peptides/proteins, the in vivo stability can be challenging as transchelation can occur resulting in unwanted high accumulation of radiocopper in the liver [44]. Several studies have demonstrated an improved in vivo stability and performance of the NOTA chelator compared with DOTA for ^64^Cu-labeling of peptides [33,45,46]. In this study, we evaluated the GRPR antagonist PEG_2_-RM26 conjugated to the macrocyclic chelators NOTA and NODAGA, labeled with [^64^Cu]Cu and compared them with radiocobalt (^55/57^Co) labeled counterparts (the latter was previously evaluated by our group with promising results [28]). We consider that the direct comparison of tracers in the same batch of tumor-bearing mice is a methodological advantage. The use of ^57^Co instead of ^55^Co allowed us to perform ex vivo biodistribution studies in dual isotope mode. This setup decreases the batch-to-batch variability of both murine physiology and target expression in cells used for tumor inoculation and should be a preferable way for preclinical comparative evaluation. Furthermore, the use of this dual-isotope approach further improves statistical power and is ethically advantageous because the number of living test subjects can be reduced, which is one of the “3R” principles of animal welfare. The risk of receptor saturation of GRPR targets should not be an issue since we used a low molar mass far from binding saturation [23,47].

The radiolabeling of NOTA/NODAGA-PEG_2_-RM26 with ^64^Cu gave products with high yield and radiochemical purity without the need for further purification. The final ^64^Cu-chelates were found to be relatively stable against transchelation with EDTA challenge. However, activity uptake in the liver was somewhat higher for [^64^Cu]Cu-NOTA-PEG_2_-RM26 conjugate than for other tested conjugates, which probably indicates a high degree of transchelation in accordance with previous findings [44].

The presence of a negative charge at the N-terminus of the ^64^Cu-labeled NODAGA-conjugate resulted in a lower affinity for [^64^Cu]Cu-NODAGA-PEG_2_-RM26 which resulted in lower uptake both in vitro and in vivo in comparison with the neutrally charged [^64^Cu]Cu-NOTA-PEG_2_-RM26. In agreement with this, the in vitro and in vivo activity retention over time was better for [^64^Cu]Cu-NOTA-PEG_2_-RM26 compared with [^64^Cu]Cu-NODAGA-PEG_2_-RM26 (Figure 3). Similarly, increased affinity for copper-labeled NOTA conjugate compared to NODAGA was also observed in a study from Gourni et al., who evaluated the GRPR-targeting antagonist MJ9 [48]. The same pattern was observed for the cobalt-labeled counterparts where the previously published data also showed the best affinity in vivo for the NOTA conjugate [28]. However, the affinity of the ^64^Cu-labeled NODAGA-conjugate was in the subnanomolar range and hence, still adequate for molecular imaging. In agreement with the in vitro data, [^64^Cu]Cu-NOTA-PEG_2_-RM26 showed a tendency to higher uptake in the tumor (*p* = 0.7) and GRPR-expressing tissues (pancreas, small intestine, and stomach) at 3 h p.i. in comparison with [^64^Cu]Cu-NODAGA-PEG_2_-RM26. However, the difference was not significant.

Comparison of biodistribution data for the ^64^Cu-labeled conjugates showed fast clearance from the blood for both conjugates, though, significantly slower for [^64^Cu]Cu-NOTA-PEG_2_-RM26. This phenomenon was associated with higher hepatic and lower renal activity uptake of the [^64^Cu]Cu-NOTA-PEG_2_-conjugate. A similar ratio between hepatic and renal excretion was observed for anti-HER2 affibody molecules labeled with radiocopper via NOTA and NODAGA chelators [49].

Already at 3 h p.i. both ^55^Co- and ^64^Cu-labeled conjugates allowed clear visualization of GRPR-expressing tumors because tumor activity uptake exceeded normal tissue uptake (Figure 6A). Tumor-to-organ ratios did not increase with time because of the rapid washout of tumor-associated activity (Figure 5). However, intensive clearance of activity in the GI tract content improved overall imaging contrast (Figure 6B). The in vivo data for ^55/57^Co-labeled RM26-based conjugates obtained in this study contradicts the data published by our group earlier for this conjugate [27], where tumor-to-organ ratios increased with time. We speculate that this could be attributed to batch-to-batch xenograft variability. This observation underlines our approach to compare ^64^Cu- and ^57^Co-labeled conjugates in the same batch of animals.

[^64^Cu]Cu-NOTA-PEG_2_-RM26 had overall the highest uptake in the liver, which was two-fold higher compared with ^64^Cu-labeled NODAGA-PEG_2_-RM26 and ten-fold higher compared with the [^57^Co]Co-NOTA-PEG_2_-RM26 at 3 h p.i. At the 24 h time-point, the activity accumulation in the liver was still significantly higher for [^64^Cu]Cu-NOTA-PEG_2_-RM26 in comparison with other tested agents. Despite the observed elevated liver accumulation for [^64^Cu]Cu-NOTA-PEG_2_-RM26, the tumor-to-liver ratio was equal in comparison to [^64^Cu]Cu-NODAGA-PEG_2_-RM26 at 3 h p.i. due to the higher tumor uptake of the NOTA analog. The observed accumulation of activity in liver tissue for [^64^Cu]Cu-NOTA-PEG_2_-RM26 is probably due to transchelation of the ^64^Cu^2+^ to serum components which circulate in the blood or to superoxide dismutase that can accumulate in liver tissue. Another process that could slow down blood clearance is off-target interactions of the probe with blood plasma proteins. This phenomenon was observed both for small molecular drugs and for proteins, and it was suggested that both lipophilic/hydrophilic and charged patches could influence these interactions [50,51]. However, the relatively high liver accumulation for [^64^Cu]Cu-NOTA-PEG_2_-RM26 at 3 h p.i. was equal or lower to other reported studies of the ^64^Cu-labeled GRPR antagonists with different chelators such as DOTHA_2_, NOTA, NODAGA, and MeCoSar evaluated in a similar animal model [46,48,52,53]. We could conclude that both [^64^Cu]Cu-NOTA/NODAGA-PEG_2_-RM26 complexes were sufficiently stable in vivo, however, less stable than their ^55/57^Co-labeled counterparts as displayed in Figure 5 and Figure 6.

As expected, our results showed that both the ^55/57^Co- and ^64^Cu-labeled peptides were predominantly eliminated through kidney excretion. However, a chelator dependent difference was also observed. The presence of an additional negative charge resulted in significantly higher kidney accumulation for [^64^Cu]Cu-NODAGA-PEG_2_-RM26 and [^57^Co]Co-NODAGA-PEG_2_-RM26 at 3 h p.i. than the NOTA-containing counterparts. At the late time-point (24 h p.i.), the observed differences in kidney uptake were evened out. The elevated kidney uptake at 24 h p.i. for [^64^Cu]Cu-NOTA-PEG_2_-RM26 could potentially be due to the slower elimination for the reasons discussed above. This slow elimination could also be a reason for the observed elevated uptake in blood at 24 h p.i. for [^64^Cu]Cu-NOTA-PEG_2_-RM26 in comparison with [^64^Cu]Cu-NODAGA-RM26 and both the ^57^Co-labeled peptides. Overall slow elimination of activity should result in higher dose to the patient. The choice of radionuclide for next day PET imaging is challenging. Among available radiometals with adequate half-lives, ^66^Ga (9.5 h), ^64^Cu (12.7 h), ^86^Y (14.7 h), and ^55^Co (17.5 h), ^64^Cu has the lowest emission of photons and the lowest energy of emitted positrons (653 keV) that should improve imaging quality. However, this isotope has the lowest positron-abundance (17.4%) among the mentioned radiometals; that would require an injection of a 2 to 4-fold higher amount of activity to get a similar signal in the PET acquisition. ^55^Co has the highest abundance of positrons (76%). It also has lower energy of emitted positrons (1498 keV) and a better ratio between emitted positrons and co-emitted gammas than for ^86^Y and ^66^Ga (all data are from [54]). More human data for radiometals with long half-lives for both distribution and imaging sensitivity are required for accurate comparison of the mentioned PET nuclides. However, based on preclinical data, we recently estimated the effective total patient dose for a [^55^Co]Co-DOTATATE scan to be 4.7 mSv, which was comparable to the effective dose for [^64^Cu]Cu-DOTATATE of 6.5 mSv [44]. This includes correction for the more than 4-fold difference in positron yield between ^55^Co and ^64^Cu to obtain the same equivalent number of annihilation events in identical PET scanners.

The significantly lower uptake in blood and liver for the ^57^Co-labeled conjugates could be the result of better in vivo stability of the cobalt–chelator complexes and/or lower degree of their off-target interactions in comparison to the ^64^Cu-labeled counterparts. This and the high tumor uptake for the ^57^Co-labeled radioconjugates, especially for NODAGA-PEG_2_-RM26, resulted in overall higher tumor-to-non-tumor ratios compared to the ^64^Cu-labeled counterparts, leading to increased image contrast. The tumor-to-background ratios, particularly for blood, intestine walls, muscle, and bone, are very important in diagnostic imaging of PC since the high contrast between malignant and normal tissue increases the detection rate. Advanced PC often metastasizes to lymph nodes, and bone, where detection of small distant metastases is crucial to obtain an accurate staging of the disease.

## 4. Materials and Methods

[^57^Co]Co-chloride was purchased from PerkinElmer (Upplands Vasby, Sweden). ^55^Co and ^64^Cu were produced in-house at Odense University Hospital as previously described [44,55]. In one instance, the ^64^Cu was purchased at DTU NuTech, Technical University of Denmark. The GRPR antagonists NOTA-PEG_2_-RM26 and NODAGA-PEG_2_-RM26 were synthesized as described earlier [26]. Buffers for radiolabeling were produced in-house from chemicals supplied by Merck (Darmstadt, Germany) and were pretreated with Chelex 100 resin (Bio-Rad Laboratories, Hercules, CA, USA) to remove metal contaminants. Other chemicals were purchased from Sigma-Aldrich Sweden (Upplands Vasby, Sweden). Radioactive samples were measured in an automated gamma-counter (2470 Wizard Automatic Gamma Counter, Perkin-Elmer).

PC-3 human PC cell line expressing GRPR was purchased from ATCC, LGC Promochem. Cells were cultured in RPMI-1640 media supplemented with 10% fetal calf serum (Sigma), PEST (penicillin 100 IU/mL, streptomycin 100 g/mL), and 2 mM l-glutamine (all from Biochrom AG, Berlin, Germany). This medium is referred to in the text as complete medium. Trypsin-EDTA (0.05% trypsin, 0.02% EDTA in buffer) was purchased from Biochrom AG.

### 4.1. Labeling and Stability

Labeling with [^64^Cu]CuCl_2_ was performed using 10 µL (1–10 nmol) of X-RM26 (X = NOTA-PEG_2_, NODAGA-PEG_2_), buffered with 100 µL of sodium acetate buffer (0.2 M, pH 5.5). After the addition of 20–65 μL of [^64^Cu]CuCl_2_ (40–100 MBq), the reaction mixture was heated by microwave irradiation for 2 min (65–75 °C) in a sealed vial using a PETWave (CEM Corporation, Matthews, NC, USA). Labeling with ^57^Co was performed using 1 nmol (aq. 0.1 nmol/µL) of X-RM26 buffered with 100 µL ammonium acetate (0.2 M, pH 5.5). [^57^Co]CoCl_2_ (2.5 MBq) was added to the mixture followed by microwave irradiation for 2 min (65–75 °C). For ^55^Co labeling, 1.3 nmol of X-RM26 buffered with 80 µL ammonium acetate (0.2 M, pH 5.5) was heated by microwave irradiation for 1 min at 850 W in a sealed vial, as previously described [24]. The radiochemical yield and purity were determined by high-performance liquid chromatography (HPLC). Samples were analyzed using instant thin-layer chromatography (ITLC) strips (Biodex Medical Systems) eluted with 0.2 M citric acid, pH 2.0. Radiometal-chelate stability was evaluated by incubation in the presence of 1000-fold excess EDTA for 1 h, at RT, and in murine serum (1 h, 37 °C).

### 4.2. In Vitro Studies

GRPR expressing PC-3 PC cells were used for in vitro studies. To evaluate in vitro binding specificity of [^64^Cu]Cu-X-RM26 to GRPR, a group of cell dishes was incubated with an excess amount (300-fold) of non-labeled peptide for 10 min at RT to block GRPR receptors prior to the addition of the radioactive solution containing [^64^Cu]Cu-X-RM26 (1 nM). After 1 h incubation at 37 °C, the cells were washed with serum-free media and detached using trypsin-EDTA solution. Measurements of cell-associated activity were done against standards in an automated gamma-counter. Cellular processing was evaluated by incubating PC-3 cells with 1 nM solution of [^64^Cu]Cu-X-RM26 at 37 °C. At predetermined time points (1, 2, 4, 8, and 24 h after the start of incubation), the membrane-bound and internalized activity were collected using the acid wash method [23]. To assess the cellular retention of activity, PC-3 cells were incubated for 1 h at 4 °C with 1 nM solution of [^64^Cu]Cu-X-RM26. The radioactive media was replaced by fresh complete media, and the cells were incubated at 37 °C. At predetermined time-points (1, 2, 4, 8, and 24 h), membrane-bound and internalized activity fractions were collected using the previously described acid wash method. The samples were measured in an automated gamma-counter. The half-maximal inhibitory concentration (IC_50_) was determined for ^nat^Cu-X-RM26 using the universal BN radioligand ^125^I-Tyr^4^-BBN (Perkin Elmer). PC-3 cells were incubated with ^125^I-Tyr^4^-BBN (0.1 pmol/well) at 4 °C for 5 h in the presence of increasing concentrations of ^nat^Cu-X-RM26 (0.5, 2, 5, 50, 200, and 600 nM). Following incubation, cells were collected and the cell-associated activity was measured in an automated gamma-counter.

### 4.3. In Vivo Studies

All animal experiments were planned and performed in accordance with the national legislation on the protection of laboratory animals, and the study plans were approved by the Animal Experiments Inspectorate in Denmark (approval number 2016-15-0201-01027).

For biodistribution studies, 16 female BALB/c nu/nu mice (age 15–16 weeks) bearing PC-3 xenografts (inoculated subcutaneously with 8 × 10^6^ PC-3 cells three weeks before the experiment) were randomized into groups of four. The mice were intravenously injected into the tail vein with a mixture of [^64^Cu]Cu-X-RM26 and [^57^Co]Co-X-RM26 and were euthanized at 3 and 24 h p.i. Injected activity was adjusted to 300 kBq/mouse for ^64^Cu- and 40 kBq/mouse for ^57^Co-labeled conjugates. The total injected peptide mass was adjusted to 45 pmol/mouse (in 100 μL). Blood, kidney, pancreas, liver, lung, bone, muscle, tumor, spleen, stomach, small intestines, and the rest of the GI tract with content were collected, weighed, and their activity content was measured in a gamma-counter. The ^64^Cu activities were determined from measurements performed on the day of the experiment, while the ^57^Co activities were measured after two weeks to allow the ^64^Cu to decay. Tissue uptake of the radiopeptides was calculated as a percent of injected dose per gram tissue (%ID/g), with exception of the GI tract for which tissue uptake was calculated as %ID per whole sample.

### 4.4. Imaging

Whole body PET/CT scans were performed using a Siemens Inveon preclinical scanner (Siemens Healthcare, Knoxville, USA) on PC-3 xenografted male NOD-scid mice (in-bread, age 12–13 weeks). The mice (*n* = 2/group) were anesthetized with a mixture of 1.5–2% isoflurane and 100% oxygen and injected via the tail vein with either 2.0–2.9 MBq (0.24–0.32 nmol) of [^55^Co]Co-X-RM26 or 3.1–3.7 MBq (0.18–0.23 nmol) of [^64^Cu]Cu-X-RM26, respectively. At 3 and 24 h p.i., the mice were anesthetized and PET/CT scanned with PET acquisition times of 15 and 30 min, respectively. All mice were awakened from anesthesia between longitudinal scans and allowed to roam freely in cages with unrestricted access to food and water. The CT scans were performed with 2 bed positions, 270 projections in 360 degrees’ rotation, and with bin 4. CT and PET images were co-registered and the CT-based attenuation corrected PET data were reconstructed using an OSEM3D/MAP algorithm (4 OSEM3D iterations, 16 MAP subsets, and 18 MAP iterations, target resolution 1.5 mm). PET and CT data were analyzed using the Inveon Research Workplace (Siemens Healthcare) and presented as maximum intensity projections (MIPs) adjusted to display a color scale from 0 to the maximum tumor uptake value in the actual scan.

### 4.5. Statistics

Data were analyzed using GraphPad Prism (version 7.03, GraphPad Software Inc., San Diego, CA, USA) by explorative statistics using one-way ANOVA with Holm-Sidak correction for multiple comparisons to determine significant statistical differences (*p* < 0.05). All p-values given are adjusted for multiple comparisons. The IC_50_ values were calculated by nonlinear regression using GraphPad Prism. In vitro data are presented as mean values including standard deviation (SD). In vivo data as mean including standard error of means (SEM).

## 5. Conclusions

In this study, we prepared the GRPR-targeting antagonists [^64^Cu]Cu-NOTA-PEG_2_-RM26 and [^64^Cu]Cu-NODAGA-PEG_2_-RM26 with high yields and high radiochemical purities. Both radiolabeled peptides showed high affinities for GRPR in vitro. Ex vivo biodistribution and PET/CT images showed high accumulation in GRPR-expressing PC-3 tumors for both conjugates resulting in favorable tumor-to-background ratios. [^64^Cu]Cu-NOTA-PEG_2_-RM26 had an overall higher uptake in non-targeted tissues resulting in decreased tumor-to-background ratios. The head-to-head comparison of ^64^Cu- and ^55/57^Co-labeled conjugates showed comparable pharmacokinetic profiles, though [^55/57^Co]Co-NOTA/NODAGA-PEG_2_-RM26 presented important advantages by significantly higher tumor-to-background ratios at the early time-points. Hence, the [^55/57^Co]Co-NOTA-PEG_2_-RM26 showed the overall best in vivo characteristics. However, all tested radioconjugates were able to visualize GRPR-expression at early and later time-points with high image contrast. These results indicate that for PET imaging, [^55^Co]Co-NOTA/NODAGA-PEG_2_-RM26 are preferred to the ^64^Cu-labeled counterparts due to the increased in vivo stability and overall higher tumor-to-non-tumor ratios, thus providing a suitable candidate for clinical translation.

## Figures and Tables

**Table 1:** Labeling and stability of [^64^Cu]Cu-X-RM26 (X = NOTA-PEG_2_, NODAGA-PEG_2_). Stability was checked in serum samples after 1 h incubation at 37 °C and in the presence of 1000× excess of ethylenediaminetetraacetic acid (EDTA) after 1 h incubation at RT. Data are presented as average ± standard deviation.

[^64^Cu]Cu-X-RM26	NOTA	NODAGA
Labeling yield (4 MBq/nmol), %	98.93 ± 0.09	98.9 ± 0.2
Release in serum (1 h, 37 °C), %	0.2 ± 0.2	0.10 ± 0.06
Release in the presence of excess EDTA (1 h, RT), %	0.8 ± 0.2	0.96 ± 1.07

**Table 2:** The biodistribution results for [^64^Cu]Cu-X-RM26 and [^57^Co]Co-X-RM26 (X = NOTA-PEG_2_ and NODAGA-PEG_2_) in BALB C/nu mice at 3 and 24 h p.i.

Organ	[^64^Cu]Cu-NOTA				[^64^Cu]Cu-NODAGA				[^57^Co]Co-NOTA				[^57^Co]Co-NODAGA			
	3 h		24 h		3 h		24 h		3 h		24 h		3 h		24 h	
Blood	0.06	±0.006 ^a,b,c^	0.04	±0.001 ^a,b,c^	0.03	±0.002 ^a^	0.02	±0.01 ^a^	0.02	±0.002 ^b^	0.01	±0.001 ^b^	0.03	±0.01 ^c^	0.01	±0.001 ^c^
Kidney	0.46	±0.04 ^a^	0.25	±0.008	0.90	±0.09 ^a,d^	0.12	±0.01	0.51	±0.08 ^d,f^	0.06	±0.02	0.84	±0.13 ^f^	0.07	±0.01
GI tract	0.76	±0.15 ^b^	0.07	±0.02	1.02	±0.15 ^d^	0.03	±0.01	0.26	±0.08 ^b,d,f^	0.02	±0.004	0.99	±0.16 ^f^	0.24	±0.22
Stomach	1.19	±0.16 ^a,b,c^	0.14	±0.03	0.27	±0.05 ^a,d^	0.08	±0.03	0.59	±0.04 ^b,d^	0.06	±0.01	0.48	±0.04 ^c^	0.04	±0.01
Spleen	0.10	±0.01 ^a^	0.05	±0.01	0.05	±0.01 ^a^	0.02	±0.01	0.07	±0.01	0.04	±0.003	0.08	±0.01	0.04	±0.003
Small int.	0.72	±0.10 ^a,b,c^	0.12	±0.01	0.24	±0.04 ^a^	0.05	±0.01	0.34	±0.06 ^b,f^	0.08	±0.02	0.12	±0.03 ^c,f^	0.04	±0.02
Pancreas	1.31	±0.33 ^a,b^	0.10	±0.02	0.27	±0.04 ^a^	0.04	±0.02	0.44	±0.05 ^b^	0.02	±0.001	0.76	±0.20	0.03	±0.001
Liver	0.59	±0.07 ^a,b,c^	0.49	±0.01 ^a,b,c^	0.28	±0.04 ^a,d,e^	0.16	±0.02 ^a,d,e^	0.06	±0.001 ^b,d^	0.01	±0.002 ^b,d^	0.07	±0.01 ^c,e^	0.01	±0.002 ^c,e^
Lung	0.21	±0.02 ^a,b,c^	0.14	±0.01 ^a,b,c^	0.12	±0.01 ^a,d,e^	0.04	±0.01 ^a^	0.05	±0.004 ^b,d^	0.02	±0.001 ^b^	0.05	±0.01 ^c,e^	0.02	±0.002 ^c^
Bone	0.07	±0.02	0.15	±0.09	0.08	±0.02	0.13	±0.00	0.11	±0.04	0.09	±0.03	0.13	±0.03	0.14	±0.01
Tumor	3.94	±0.36	0.42	±0.04	2.31	±0.41	0.16	±0.04	3.27	±1.07	0.31	±0.04	4.58	±1.56	0.50	±0.21
Muscle	0.02	±0.003	0.01	±0.0000	0.02	±0.01	0.00	±0.0000	0.02	±0.002	0.01	±0.002	0.02	±0.01	0.01	±0.002

The organ uptake values are expressed as a percentage of the injected dose per gram of tissue weight (%ID/g), except for the gastrointestinal (GI) tract, for which the values were expressed as a percentage of the injected dose per sample (%ID). All values are presented as mean ± standard error of mean (SEM). Significant difference (*p* < 0.05) at the same time point: ^a^ between [^64^Cu]Cu-NOTA and [^64^Cu]Cu-NODAGA; ^b^ between [^64^Cu]Cu-NOTA and [^57^Co]Co-NOTA; ^c^ between [^64^Cu]Cu-NOTA and [^57^Co]Co-NODAGA; ^d^ between [^64^Cu]Cu-NODAGA and [^57^Co]Co-NOTA; ^e^ between [^64^Cu]Cu-NODAGA and [^57^Co]Co-NODAGA; ^f^ between [^57^Co]Co-NOTA and [^57^Co]Co-NODAGA.

## Supplementary Materials

The following are available online, Figure S1: Biodistribution of [^64^Cu]Cu-X-RM26 (X = NOTA-PEG_2_, NODAGA-PEG_2_) in PC-3 xenografted BALB/C nu/nu mice at (A) 3 h and (B) 24 h p.i. The organ uptake values are expressed as a percentage of the injected dose per gram of tissue weight (%ID/g) except the gastrointestinal (GI) tract for which the values were expressed as a percentage of the injected dose per sample (%ID); Table S1: Tumor-to-organ ratios for biodistribution data.
