# Supplementary material for: Preclinical Evaluation of the Copper-64 Labeled GRPR-Antagonist RM26 in Comparison with the Cobalt-55 Labeled Counterpart for PET-Imaging of Prostate Cancer
Source: Molecules. 2020 Dec 18;25(24):5993. doi: 10.3390/molecules25245993 (PMC7766840; doi:10.3390/molecules25245993)
Supplement: Supplementary file 1 [file molecules-25-05993-s001.pdf]

Table S1. Tumor-to-organ ratios for biodistribution data

	$[^{64}Cu]$Cu-NOTA		$[^{64}Cu]$Cu-NODAGA		$[^{57}Co]$Co-NOTA		$[^{57}Co]$Co-NODAGA	
Tumor/organ	3 h	24 h	3 h	24 h	3 h	24 h	3 h	24 h
Blood	66.96 ± 15.46	10.88 ± 2.68	83.09 ± 30.36	10.54 ± 9.04	259.72 ± 203.22	62.17 ± 19.02	203.62 ± 128.57	91.65 ± 98.90
Kidney	8.91 ± 2.43	1.69 ± 0.34	2.43 ± 0.99	1.34 ± 0.65	7.26 ± 5.66	6.05 ± 3.86	6.34 ± 6.25	8.41 ± 8.73
GI	5.66 ± 2.15	6.27 ± 2.11	2.30 ± 0.60	5.90 ± 1.96	18.30 ± 12.63	15.30 ± 7.07	5.29 ± 4.38	14.27 ± 14.38
Stomach	3.60 ± 1.67	3.13 ± 1.02[c]	9.08 ± 2.12	2.45 ± 1.17[e]	5.87 ± 4.24	5.31 ± 1.53[c]	9.14 ± 5.48	13.74 ± 9.32[e]
Spleen	43.62 ± 17.16	12.19 ± 10.60	45.38 ± 7.95	11.29 ± 7.06	62.80 ± 60.60	7.77 ± 1.55	61.25 ± 36.71	11.79 ± 8.29
Small int.	5.71 ± 1.42[c]	3.76 ± 1.50	9.92 ± 2.55	3.82 ± 2.37	11.45 ± 9.57	4.55 ± 2.11	47.92 ± 40.36[c]	18.46 ± 17.33
Pancreas	3.45 ± 1.41	4.77 ± 2.28	8.90 ± 2.83	5.49 ± 3.99	7.90 ± 5.28	12.67 ± 2.71	6.55 ± 3.84	19.32 ± 16.52
Liver	6.91 ± 1.62[b,c]	0.86 ± 0.15	8.42 ± 2.42[e]	1.02 ± 0.51	56.54 ± 37.11[b]	24.37 ± 5.29	68.36 ± 36.19[c,e]	33.95 ± 20.39
Lung	18.98 ± 3.67[c]	3.09 ± 1.18	19.38 ± 4.53[e]	6.37 ± 4.33	75.74 ± 55.74	14.64 ± 4.27	89.42 ± 51.52[c,e]	22.53 ± 22.28
Bone	100.32 ± 98.50	6.50 ± 4.41	35.31 ± 19.12	0.84 ± 0.00	43.85 ± 33.41	4.58 ± 3.62	44.88 ± 42.93	3.85 ± 4.29
Muscle	277.32 ± 91.62	56.65 ± 0.00	557.15 ± 634.61	62.58 ± 0.00	208.90 ± 138.41	28.24 ± 11.04	225.11 ± 161.38	43.95 ± 27.26

Supplementary table 1. Tumor-to-organ ratios for $[^{64}Cu]$Cu-X-RM26 and [57Co]Co-X-RM26 (X= NOTA-PEG_2 and NODAGA-PEG_2) in BALB C/nu mice at 3 h and 24 h p.i. All values are presented as mean ±standard error of mean (SEM). Significant difference ($p<0.05$) at the same time point:

[a] between $[^{64}Cu]$Cu-NOTA and $[^{64}Cu]$Cu-NODAGA; [b] between $[^{64}Cu]$Cu-NOTA and $[^{57}Co]$Co-NOTA; [c] between $[^{64}Cu]$Cu-NOTA and $[^{57}Co]$Co-NODAGA; [d] between $[^{64}Cu]$Cu-NODAGA and $[^{57}Co]$Co-NOTA; [e] between $[^{64}Cu]$Cu-NODAGA and $[^{57}Co]$Co-NODAGA; [f] between $[^{57}Co]$Co-NOTA and $[^{57}Co]$Co-NODAGA.

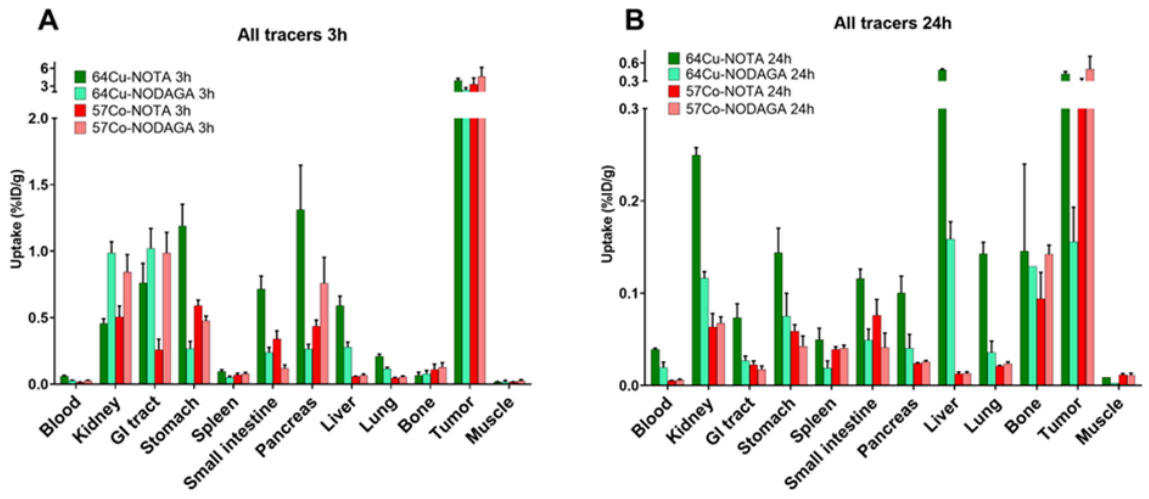

Figure S1: Biodistribution of [^{64}Cu]Cu-X -RM26 (X=NOTA-PEG_2, NODAGA-PEG_2) in PC-3 xenografted BALB/C nu/nu mice at (**A**) 3 h and (**B**) 24 h p.i. The organ uptake values are expressed as a percentage of the injected dose per gram of tissue weight (%ID/g) except the gastrointestinal (GI) tract for which the values were expressed as a percentage of the injected dose per sample (%ID).
